# Sustainable Behavior Change for Health Supported by Person-Tailored, Adaptive, Risk-Aware Digital Coaching in a Social Context: Study Protocol for the STAR-C Research Programme

**DOI:** 10.3389/fpubh.2021.593453

**Published:** 2021-03-01

**Authors:** Nawi Ng, Malin Eriksson, Esteban Guerrero, Carina Gustafsson, John Kinsman, Jens Lindberg, Helena Lindgren, Kristina Lindvall, Anna Sofia Lundgren, Göran Lönnberg, Klas-Göran Sahlen, Ailiana Santosa, Linda Richter Sundberg, Lars Weinehall, Patrik Wennberg

**Affiliations:** ^1^Department of Epidemiology and Global Health, Faculty of Medicine, Umeå University, Umeå, Sweden; ^2^School of Public Health and Community Medicine, Institution of Medicine, Sahlgrenska Academy, University of Gothenburg, Gothenburg, Sweden; ^3^Department of Social Work, Faculty of Social Sciences, Umeå University, Umeå, Sweden; ^4^Department of Computing Science, Faculty of Science and Technology, Umeå University, Umeå, Sweden; ^5^Public Health Unit, Region Västerbotten, Umeå, Sweden; ^6^Department of Culture and Media Studies, Faculty of Arts and Humanities, Umeå University, Umeå, Sweden; ^7^Department of Public Health and Clinical Medicine, Faculty of Medicine, Umeå University, Umeå, Sweden

**Keywords:** behavioural change, digital coaching, interdisciplinary programme, formative research, evaluation of intervention, social network, social media, health behaviour trajectories

## Abstract

**Introduction:** The Västerbotten Intervention Programme (VIP) in the Region Västerbotten Sweden is one of the very few cardiovascular disease (CVD) prevention programmes globally that is integrated into routine primary health care. The VIP has been shown as a cost-effective intervention to significantly reduce CVD mortality. However, little is known about the effectiveness of a digital solution to tailor risk communication strategies for supporting behavioral change. STAR-C aims to develop and evaluate a technical platform for personalized digital coaching that will support behavioral change aimed at preventing CVD.

**Methods:** STAR-C employs a mixed-methods design in seven multidisciplinary projects, which runs in two phases during 2019–2024: (i) a formative intervention design and development phase, and (ii) an intervention implementation and evaluation phase. In the 1st phase, STAR-C will model the trajectories of health behaviors and their impact on CVDs (Project 1), evaluate the role of the social environment and social networks on behavioral change (Project 2) and assess whether and how social media facilitates the spread of health information beyond targeted individuals and stimulates public engagement in health promotion (Project 3). The findings will be utilized in carrying out the iterative, user-centered design, and development of a person-tailored digital coaching platform (Project 4). In the 2nd phase, STAR-C will evaluate the implementation of the coaching programme and its effectiveness for promoting behavioral change and the spreading of health information across social networks and via social media (Project 5). The cost-effectiveness (Project 6) and ethical issues (Project 7) related to the coaching programme intervention will be evaluated.

**Discussion:** The STAR-C research programme will address the knowledge and practice research gaps in the use of information technologies in health promotion and non-communicable disease (NCD) prevention programmes in order to narrow the health inequality gaps.

**Ethics:** STAR-C has received approval from the Swedish Ethical Review Authority (Dnr. 2019-02924;2020-02985).

**Dissemination:** The collaboration between Umeå University and Region Västerbotten will ensure the feasibility of STAR-C in the service delivery context. Results will be communicated with decision-makers at different levels of society, stakeholders from other regions and healthcare professional organizations, and through NGOs, local and social media platforms.

## Introduction

Chronic non-communicable diseases (NCDs) are the leading burden of disease globally; responsible for about two-thirds of all deaths (1). Even though cardiovascular disease (CVD) morbidity and mortality rates in Sweden have decreased in the last few decades, CVDs remain the main cause of premature deaths (< age 75 years) in women and men (2). Although evidence for the effectiveness of lifestyle modification and pharmacological treatment among high-risk individuals is well-established (3), these high-risk approaches have failed to show significant reductions in CVD morbidity and mortality at population level (4), hence their effectiveness is often questioned. Since 60–70% of CVD events occur among individuals with only moderate risk, CVD prevention strategies should ideally target the whole population, including the large group with moderate CVD risk (5). This group is typically under-represented in existing CVD prevention interventions (3), thus denying the individuals with moderate CVD risk the possibility of benefitting from the interventions. Multi-sectoral, population-wide interventions can form a strategy for reaching these groups, while also acting as a means of striving toward the principles of “fair society and healthy lives.” These principles comprise the essence of the message from the WHO Commission on Social Determinants of Health (6). These population-wide interventions could be essential in reducing premature mortality from NCDs, which constitutes one of the targets of the Sustainable Development Goals (SDG) 3: that of ensuring healthy lives and promoting well-being for all at all ages (7).

Research to predict the future risk of NCD morbidity and mortality based on early life childhood predictors (8, 9) or well-established NCD risk factors at adult age such as smoking, obesity, and hypertension (10) have grown in the last few decades. Very few current estimates incorporate information on health behaviors' change and their determinants over the life course (8). Neither do they account for different NCD risk factors' trajectories over time (10). Moreover, many of these estimates are generic, and adaptation is not easily done in clinical settings. This limits their usability for identifying target individuals for prevention programmes. The ongoing expansion of “big data” and the advancement of tools such as machine learning and artificial intelligence in analyzing complex and unstructured data provide the possibility for the personalisation and tailoring of such risk estimations at individual level (11, 12).

The Västerbotten Intervention Programme (VIP), situated in Northern Sweden, is one of very few long-term CVD prevention programmes in the world that (1) is integrated into routine primary health care (PHC) settings, (2) targets selected age-groups in middle-age, and (3) combines low-risk population and high-risk individual strategies (13). In response to the high CVD mortality rate in the 1980s, the Region Västerbotten (RV), previously known as the Västerbotten County Council, designed and piloted a population-based health promotion programme in Norsjö Municipality. The programme combined individual and population-based strategies with multi-sectoral approaches in collaboration with the food industry and mass media, as well as health examination and health dialogue with trained district nurses at PHC centers. The programme was later scaled up to all municipalities in the county during 1990–1992 and has since been known as the VIP, integrated into routine services at 40 Västerbotten PHCs.

**Figure 1:**
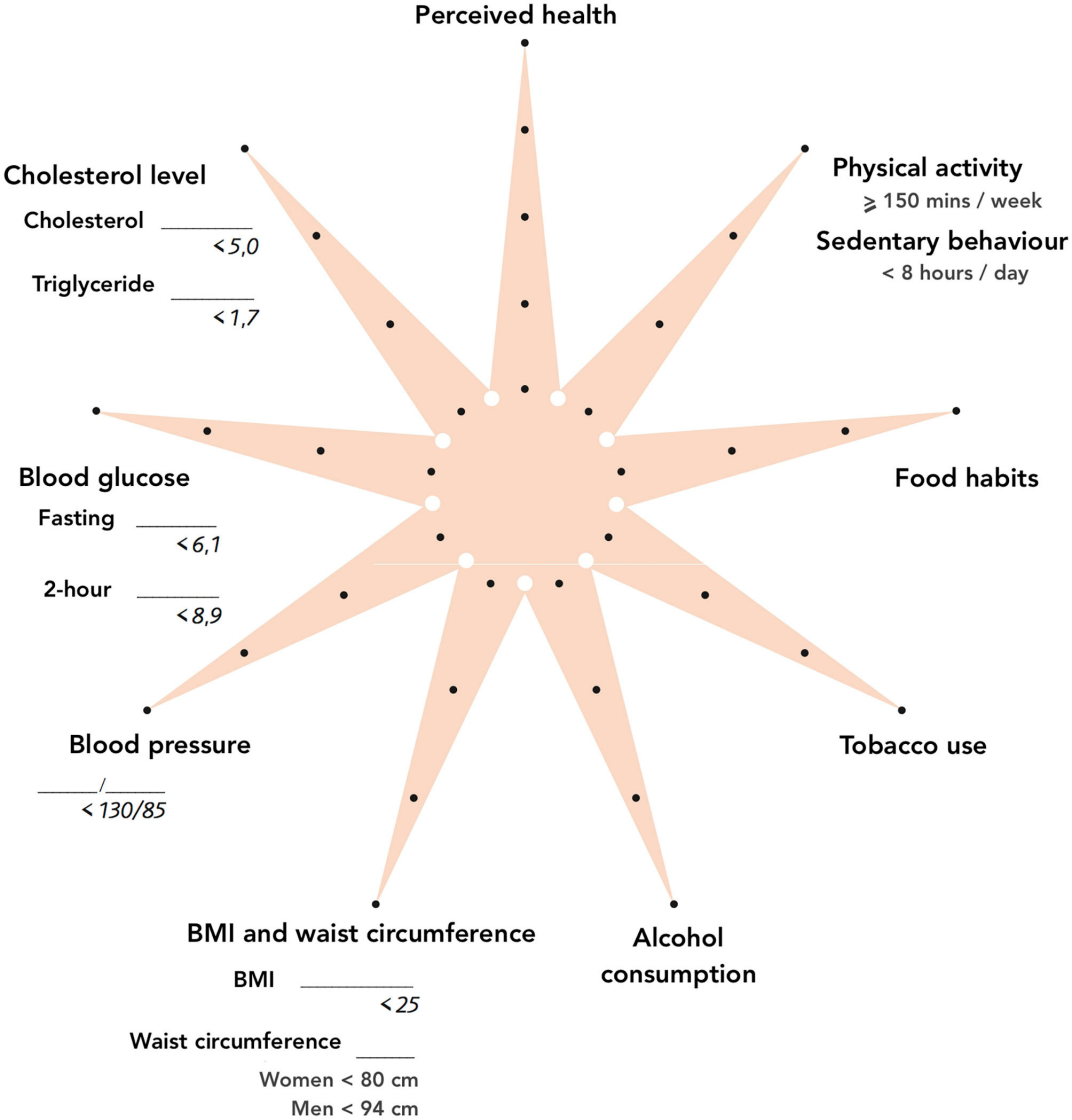
The VIP star-shaped infographic.

The VIP invites all county residents turning 40, 50, and 60 years old to a health examination that screens for CVD risk factors. In a health dialogue, the VIP nurses discuss the results of the health examinations with the participant, presenting the results in a star-shaped infographic (Figure 1). The nurses use motivational interviewing techniques to discuss strategies for the adoption of healthier behaviors (14, 15). Individuals who are identified as at high risk are then referred to physicians for further evaluation and treatment. During 1990–2019, a total of 119,860 individuals participated in the VIP, with 51,025 and 13,891 individuals participating twice and thrice, respectively. A 17-year evaluation of the VIP showed a significant reduction in overall and CVD-specific premature mortality in the Västerbotten population (10%), especially among those who participated in the VIP (33%). However, the evaluation also suggested that the largest absolute number of prevented deaths occurred among people with a shorter education history and more prevalent risk factors (16). The evaluation cannot confirm whether differences in VIP's adoption by PHCs or differences in VIP recommendation uptakes by the population in the different socio-economic groups could explain the heterogeneity of the VIP impacts observed.

A few studies have evaluated factors related to participation in the VIP (13, 17), the effect of participation in the VIP on self-rated health and risk factor load (18) and on mortality (16) as well as strategies in delivering health-promoting dialogues by the VIP nurses (19). However, there is currently limited knowledge on strategies to reach those that the VIP has not yet reached, on the best communication strategies regarding behavioral risks, on the factors that affect the participant's adoption of the VIP's health promoting messages, as well as on factors related to health providers that affect VIP implementation at different PHCs. The use of communication technology between the participants, the nurses and beyond in the VIP are still in their infancy. Neither do we know how information related to the VIP spreads beyond the participants in their social networks; an effect of which can be the amplification of the intended VIP impact within the population. When the VIP was launched in Västerbotten County in 1985, associations, newspapers, radio and television, and the food industry played critical roles in raising community awareness about NCDs and stimulating public debate (13). However, these population components of VIP have gradually diminished in the last three decades. The growth of online information and social media has changed the roles that associations and collective activities once played in the VIP, and this new, social network dynamic calls for a new approach in providing support for behavioral change in the community.

Previous research has shown that gender influences risk perception, risk communication and health behavior change. Previous research suggests that men are more likely than women to underestimate health risks related to adverse health behaviors. Furthermore, men were more prone to report subjective invulnerability ideas about negative health behaviors such as smoking, drinking, and illegal drug use (20, 21). In the same lines, studies show that women, compared to men, perceive their health chances as significantly lower (22). In a study of gender differences in readiness for behavior change for stroke risk, more women were ready for behavior change to reduce stroke risk (23).

The “technological gender gap” indicates that gender influences and explains technology acceptance and adoption (24, 25), though recent findings are inconclusive (26). For example, some studies show that men are more positive and have higher adoption levels of health technology, such as mobile health devices (27). A review of technology usage and intention to use technology showed heterogeneity of gender's role and influences on using technology in different contexts (26, 28).

Knowledge of the barriers to and facilitators for implementation and adoption of a complex intervention programme supported by digital technology in routine PHC settings can be used to enhance the acceptability of the intervention, facilitate uptake and adoption, spread information about the VIP beyond the participants, and, ultimately, contribute to better and sustainable impacts of the intervention in the population at large. Our research programme (*S*ustainable behavior change for health supported by person-*T*ailored, *A*daptive, *R*isk-aware digital *C*oaching; or **STAR-C**) will address these gaps and generate new knowledge which can directly contribute to the creation of digital person-centered tools: tools aimed at improving the health promotion and NCD prevention programme, both in healthcare settings and in the general population.

### Aim and Objectives

The overall aim of STAR-C is to design and evaluate a novel, personalized digital coaching programme to promote healthy behaviors for chronic NCD prevention at individual and population levels in Northern Sweden.

STAR-C addresses seven interrelated specific objectives, as indicated below:

1. To model the trajectories of health behaviors and their impacts on health risk and outcomes in an adult population (Project 1);
2. To evaluate the role of the social environment and social networks on behavioral change (Project 2);
3. To assess whether and how social media facilitates the spread of health information beyond targeted individuals, and stimulate public engagement in health promotion (Project 3);
4. To carry out the iterative, user-centered design, and development of a platform for person-tailored digital coaching, aiming at behavioral change, and support for maintaining healthy behavior (Project 4);
5. To evaluate the implementation of the coaching programme and its effectiveness for promoting behavioral change and the spreading of health information across social networks and through social media (Project 5);
6. To analyse the cost-effectiveness of the coaching programme (Project 6); and
7. To assess ethical issues related to the collection and sharing of health-related personal data using technology platforms (Project 7).

## Methods

### Recruitment

Using the VIP as the platform, STAR-C invites VIP participants in 2020 to participate in a cross-sectional survey to measure their beliefs about their behaviors, perceived benefits of and barriers to behavioral change, readiness and stage of behavioral change, as well as their attitudes toward the use of social networks and social media for behavioral change. STAR-C will also recruit the participants and the VIP nurses from selected PHCs to further explore the core concepts of social networks and social media in relation to behavioral change and maintenance of healthy behavior using interviews and focus group discussions. The PHC will be selected to represent both rural and urban areas as well as smaller and larger municipalities in the region, bearing in mind the different health patterns in these different areas of Västerbotten. The VIP nurses will be selected/sampled to represent a variation in the geographical area described above to select PHCs and their experience in working within the VIP and conducting VIP health dialogues. The VIP participants will also vary in gender, educational level, and geographical residence (urban/rural). Selected VIP participants and nurses will also be involved in every step of developing the digital coaching platform for behavioral change.

### Study Design

STAR-C will employ a mixed method design in two phases: (i) the formative intervention design and development phase during 2019–2022; and (ii) the intervention implementation and evaluation phase during 2022–2024. STAR-C consists of seven interconnected and interdisciplinary projects that engage researchers, health professionals, and the population closely throughout its lifetime. STAR-C uses gender, equity, and ethical lenses in all phases of the programme.

### Phase 1: Formative Intervention Design and Development Phase (Year 1–4)

#### Project 1: Modeling of Behavioral Trajectories and their Impacts

This project aims to build behavioral trajectory and NCD prediction models which could be used to identify individuals at low and medium risk who could benefit from a health promotion and disease prevention programme. We will address the following questions:

1. What are the different trajectories of risk behaviors and their determinants among VIP participants?
2. Do the behavioral trajectories predict NCD morbidity and mortality?
3. What is the level of readiness and stages of change among the VIP participants to change their behaviors (among those reported to have health-risk behaviors) and to maintain their behaviors (among those reported to have health-promoting behaviors)?

##### Design

For the study on trajectories, we will use the EDVIN database, which links the individual-level VIP data with data from Statistics Sweden's LISA database (including gender, year of birth, education, and disposable income), as well as data from the Swedish National Board of Health and Welfare (including prescription register, inpatient hospitalization register, and death register data) during 1990–2017. VIP invites all individuals to participate in the health examination when they turn 40, 50, and 60. The EDVIN database contains data for 10- and 20-year follow-up of individuals who first participated in the VIP at age 40 years old. This panel data availability allows us to evaluate the trajectories of health behaviors over time in predicting NCD morbidity and mortality (10). We will also conduct a cross-sectional survey among the VIP participants in 2021 to measure their readiness for behavioral change, as well as their attitude toward the use of social networks and social media for behavioral change and maintenance of healthy behaviors. The construction of the survey is theory-driven, mainly based on the health belief model, transtheoretical model of change (29, 30), social network theory (31), and the diffusion of innovation theory (32). As the VIP data collection was put on-hold in 2020 due to Covid, we run a national-level online survey in Sweden using the same survey instrument with recruitment through Facebook and Instagram. Lesson learnt from the online survey will be used to revise the VIP survey instrument.

##### Analysis

We will conduct generalized latent class growth modeling (LCGM) to capture latent groups of behaviors and their trajectories over time and across different VIP cohorts. LCGM is a powerful statistical approach that captures the heterogeneity of changes over time (33). Using multilevel multinomial logistic regression, we will examine the associations between the trajectories and covariates, including socioeconomic and demographic factors, family history of premature NCDs, and social networks. We will implement machine-learning to build predictive models of behavioral change and NCD morbidity and mortality. We will assess the stages of change and readiness for behavior change for the VIP participants with different patterns of behavior in socioeconomic groups. All analyses will be sex stratified.

#### Project 2: The Role of Social Environment and Social Networks in Behavioral Change

This project aims to theorize about the interaction between behavioral change and social networks. The project will increase our knowledge about how gender might influence social network patterns, the gains and returns of social network involvement, and the influence of social networks on behavioral change for men and women. Previous research has indicated that the supportive effects of family social networks might be gendered in favor of men since women are often expected to be the primary provider of support to other family members (34). Studies have also found that women's supportive networks tend to go beyond family ties (i.e., friends), while men's supportive networks tend to be family-based, not least by their spouses (35). We will address the following questions:

1. What are the roles of social environments and social networking for behavioral change, maintenance of healthy behavior, and overall health?
2. What roles do social networks play in influencing behavior among individuals with different experiences of changing or maintaining behavior?
3. Are the effects of social network on behavioral change gendered? Are they different across urban/rural populations and among those with a history of shorter/longer periods within education?

##### Design

We will invite VIP participants to interview and focus-group discussion (FGD). The in-depth interviews will explore men's and women's experiences of if and how they managed to change their behaviors in a life-course perspective. The FGDs will explore norms and attitudes about the role of social networks for behavioral change. Based on preliminary results of the interviews, we will construct statements about the role of social networks and environments for behavioral change. FGD participants will then be asked to discuss these statements.

##### Analysis

Data will be analyzed using Grounded Theory situational analysis (36). We will use an open coding process to create analytical situational maps of the elements involved. The results will be illustrated in a “positional map” to lay out the different positions presented by the data on the role of social networks for behavioral change among men and women.

#### Project 3: The Role of Social Media in Behavioral Change and Health Promotion

Project 3 explores how information-sharing on social media is understood and practiced, and what it is thought to deliver. Research questions comprise:

1. How do VIP participants and VIP nurses perceive the potential role of social media for health promotion in terms of: (a) sharing health practices and results, and (b) being a source of influence through the sharing practices of peers?
2. What kind of VIP health information is shared on social media platforms and what are VIP participants' experiences of this?

##### Design

We employ qualitative ethnographic design. VIP participants and VIP nurses are invited to individual in-depth interviews to elaborate upon experiences and their general thoughts on sharing health data on social media. Interviews are semi-structured so as to open up for interviewees' own reflections when these are not covered by the interview questions. When approved by the VIP participants, interviews will include observations of the participants' social media profiles for examples of how information about the VIP is disseminated and responded to.

##### Analysis

The analysis will be based on discourse theory (37), defining discourse as a temporary configuration of meaning within a specific domain, structured through the constitutive articulation of disparate elements, including material but also symbolic and affective elements (38), such as specific wordings or emotional expressions. Interviews will be analyzed by identifying dominant discourses, including the articulation of central nodal points (privileged signs within a discourse) and conflict lines within and between discourses. Analyses are sensitive to intersections of gender, age, and geographic space (urban/rural) at work in the data. The interfaces of the applications used by the participants will be analyzed technographically with an interest in the assumptions that are encapsulated in and actuated by a particular software (39). Analysis is directed toward at the co-construction of normative ideals such as “health” between users and the technologies. Attitudes toward different modes of sharing data are explored for how they are made meaningful in relation to achieving health.

#### Project 4: Design and Development of the Personalized Digital Coaching Programme for Behavioral Change

Results from Projects 1–3 will inform the work in Project 4. The following questions will be addressed.

1. What roles do users play in the hands-on design and development of the digital technology system?
2. How can the digital technology system capture conflicting motives for behavior change, including underlying social norms and effectively support the individual in their pursuit of behavior change?
3. How can the digital system's automated learning, reasoning and decision making become transparent to the user?
4. What features in the coaching system's user interface can promote motivation, interest, and behavior change?

##### Design

Project 4 will involve the iterative, participatory, user-centered design and development of a technical platform for person-tailored digital coaching for behavior change (40). The project takes as its starting point the VIP data and takes the VIP participants as potential users. Design principles for persuasive technology and Behavior Change System design will be elaborated on in FGDs (41–44). In particular, aspects regarding motivation (29, 44), social norms, transparency, ethical, and social factors of AI-based technology will be addressed. Co-design groups will be formed, where early prototypes will be tested and modified by users, including VIP participants, nurses, and representatives of the general population. The period of iterative and formative co-design and evaluation will involve a stepwise increase of participants, who will evaluate and verify earlier design choices. An intelligent machinery will be developed where a combination of novel artificial intelligence methods will manage conflicting and changing motives, person-tailored support including risk predictions, and explanations for behavioral change.

We anticipate the end architecture will consist of three main interconnected and complementary modules: (i) The person-tailored coaching application (STAR-C), most likely as a mobile application that can be downloaded from Appstore and/or Google's store; (ii) The public risk calculator (STAR-R), possibly as a web page with the risk calculator engine, and the possibility of filling in anonymous behavioral data. Such a webpage can be embedded in/proliferated through Facebook; and (iii) the therapy version (STAR), embedded in RV's systems, which at least contains the digitalised VIP information.

### Intervention Implementation and Evaluation Phase (Year 4–6)

#### Project 5: Evaluation of the Implementation of the Personalized Digital Coaching Programme

Project 5 will implement and evaluate the technologically supported personalized digital coaching programme in a controlled trial setting. It will address the following questions:

1. Is the programme effective in promoting behavior change in the adult population? Does the effectiveness differ between men and women and between urban/rural populations and people with shorter/longer education histories?
2. What are the experiences and actual uses of the technology? How does the technology support the dissemination of the behavioral change-related activities across the individual's social network?
3. What are the barriers and facilitators for adoption of the technology, and the concerns about the technology?

##### Design

We will conduct a rigorous two-arm cluster randomized controlled trial (cRCT) design to evaluate the effectiveness of the coaching programme. In the intervention arm, the VIP participants will receive the current standard VIP health dialogue with the nurse and the personalized digital coaching programme (STAR-C platform plus external sensor devices to capture activity data). In the control arm, the participants will receive the current standard VIP health dialogue only. The primary outcomes are readiness for behavioral change, reported changes in health behaviors or maintenance of healthy behaviors, and the sharing of VIP information within social networks and in social media. In the intervention arm, we will also assess user acceptance and patterns of use of the technological platforms. Participants will be followed up through telephone interviews to measure the primary outcomes through surveys in Months 3 and 6 following their recruitment. FGDs and in-depth interviews will be conducted to explore how individuals adopt and sustain behavior change, actual use, and perceived barriers in using the platform, as well as the influence of social networks and social media on their behavior.

##### Analysis

We will use an intention-to-treat approach in the analysis of the cRCT data. A multilevel regression will be used to analyse the repeated measurements from individual participants and assess if any significant differences in the outcomes between the intervention groups exist. We will analyse social media content and activity, use of health-related terms, and information sharing based on data from the repeated quantitative surveys. We will also analyse social network patterns and how VIP-related information is being spread within the network of individuals. Interview and FGD data will be analyzed following the procedures as described in Projects 2 and 3.

#### Project 6: Cost-Effectiveness Analysis of the Personalized Digital Coaching Programme

This project aims to evaluate the distribution of health outcomes and associated costs across different population groups and for different time periods in the newly developed coaching programme, as compared to the regular VIP programme. We will address the following questions:

1. Is the personalized digital coaching for behavioral change cost-effective in promoting behavioral change as compared to the existing VIP programme only?
2. Does the cost-effectiveness differ between population groups in urban/rural areas, those with shorter/longer education histories, and those with chronic NCDs (such as diabetes or hypertension)?

##### Design

We will conduct a cost-effectiveness and a distributional cost-effectiveness study using a lifetime perspective and with a societal approach. In measuring the effectiveness of the intervention, we will use the primary and secondary outcomes as described in Project 5. Information on costs of interventions will be collected throughout the programme, focusing on the differences in costs between the two arms in Project 5. Costs will be assessed based on interviews and the regions accounting.

##### Analyses

The cost-effectiveness analysis will use the information on the costs and outcomes, using short-term and long-term perspectives. We will assess the costs for the intervention with a micro-costing approach. We will employ a Markov model based on possible events such as heart conditions, diabetes, or stroke to assess costs from a long-term perspective. Published data will guide the ascertainment of the probability of events that affects costs. Utility outcomes are collected to calculate gained QALYs in the two arms described in project 5. This project will use a probabilistic Markov model as a statistical tool to compare the population groups who have and have not changed behavior. We will use a discount rate of 3% and perform sensitivity analyses with 0 and 5%. Altogether these results will give decision-makers a tool to decide on the implementation process and the prioritization process.

#### Project 7: Evaluation of Ethical Issues Related to the Collection and Sharing of Health-Related and Personal Data Using Technology Platforms

This project aims to contribute to the discourse of ethics surrounding collection and sharing of personalized data in a research and routine primary care setting as well as the use of a digital/technological platform for behavioral change. We will contribute to the discussion of best practices to address the ethical use in using technology platforms for behavior change.

##### Design

A longitudinal qualitative study will be conducted using FGDs with VIP participants, and in-depth interviews with VIP nurses and managers. Respondents will be followed up and met three times over the course of the project (Year 2, 3, and 5). The FGD and interview data collection tools will follow a similar format. In the first round of meetings, two scenarios will be presented, concerning ethical issues that could potentially arise through the use of the technology-assisted behavioral change coaching programme. One of the scenarios will focus on issues to do with informed consent and data ownership (the bioethical principle of autonomy); the other will be concerned with understanding—and any potential misunderstandings—of the digital output, and the possible health consequences thereof (the bioethical principle of non-maleficence). Rounds 2 and 3 will focus more on specific experiences that people (participants and health care providers) have had with the technologies. FGDs and interviews will be recorded digitally, and transcribed verbatim.

##### Analyses

We will conduct a thematic analysis (45, 46). A code manual will be developed for the FGDs and the qualitative interviews. This will initially include a set of a priori codes based on, for example, ethical concerns and suggested solutions, and perceived opportunities and benefits of the apps, but with additional codes subsequently included as and when they are identified inductively during initial data readings. Transcripts of all the FGDs and interviews will then be coded accordingly, and core emerging themes will be identified. Gender, geography, and age will be taken into account in the analyses.

## Ethics and Dissemination

### Ethical Consideration

STAR-C has received an approval from the Swedish Ethical Review Authority (Dnr. 2019-02924;2020-02985). Information regarding STAR-C and its project will be provided to the study participants and written informed consent will be obtained prior to enrolment to the survey or recruitment to the interviews or focus group discussions. Since the risks of participation in the quantitative and qualitative sub-studies are considered very low, we estimate that the gains described clearly outweigh the risks.

### Confidentiality, Data Management, and Handling

Data will be handled and stored in accordance with the EU General Data Description Regulation 2016/679. The material will also be handled in accordance with the Archives Act and the document management plan that applies to research material at Umeå University. A steering group of researchers in STAR-C will review analysis plans and requests for data extraction. Surveys are completed digitally or scanned optically by ITS, Umeå University. The EDVIN database is updated via Statistics Sweden's collaboration. All quantitative data is anonymised before the research team gains access to the data. Quantitative data is stored securely in the server at the Department of Epidemiology and Global Health, Umeå University.

All transcribed interviews and FGDs are coded, so that no personal data is in the transcribed texts. The code key is stored separately from the transcribed texts. The audio files will be used to cross-check the transcribed texts and then archived in the Umeå University Archives. The transcribed texts are stored in password-protected computers during the programme time and then archived in the Umeå University archives for 10 years. Only researchers who analyse the qualitative data will have access to it. The informants will be assured that data will be anonymised and encrypted.

Data collected with prototypes will be stored securely at the Department of Computer Science, Umeå University. To use prototypes, the researcher gets a user account linked to an email address. Secure login via https is used. The username, password, and e-mail address are encrypted and stored in a database. An identifier is created by the system and used in another database where encoded data from the use of prototypes is stored, tied to the individual user. A third database is used for the content of programmes developed with identifiers for this information and is used for the encoded information. Only responsible research engineer has access to password reset functions. Researchers in STAR-C will have access to coded data. Some data is temporarily stored locally on the research subjects' mobile phones and later sent to the server.

### Dissemination

STAR-C brings together experts within academic settings, health professionals, decision-makers and citizens in Region Västerbotten (RV), alongside national stakeholders and international collaborators. The added value in this research programme is the creation of a joint collaborative space between well-established multidisciplinary research teams from different faculties at Umeå University, which will enrich innovations and discussions ongoing in the fields of technology, health promotion and disease prevention, as well as wider dissemination of the research findings, which could not have been achieved within a single-discipline research project.

The strong collaboration between Umeå University and RV constitute a great strength that will ensure the feasibility of our programme in the service delivery context, as well as the relevance of the research and its end products for the local population's needs. As a formal collaborator, RV will influence the direction of the research programme. As RV is the owner and manager of the VIP, the findings of STAR-C have the potential to make a significant impact on policy at county level. STAR-C will address several risk factors common not only to CVD, but also to diabetes, cancer, dementia, osteoporosis, etc., hence extending the benefit of the VIP beyond reducing the burden of CVD morbidity and mortality (47). Our programme will bring innovation and facilitate the diffusion of technology in dealing with the growing proportion of the population living with NCDs, as well as the currently limited availability of human resources for health promotion. The results are expected to be disseminated and have direct implications for the applications of technologies in health promotion within routine primary care services and beyond.

This project has significant potential national impacts, since CVD prevention programmes based on the same concept as VIP are ongoing or under development in seven other Swedish counties. As this project is built onto the existing VIP collaborations in the county, it has a strong basis for guiding a more effective implementation of an improved intervention programme in the future, and for dissemination of the research results. Results will be communicated with all VIP staff, decision makers at different levels of society, and stakeholders from other counties through the healthcare professional organizations such as the Swedish Health-Promoting Health (HPH/HFS in Swedish) network. Dissemination to the general population will be conducted through articles in local media, public meetings with NGOs and municipalities, and through social media platforms.

## Discussion

### Anticipated Results

STAR-C is an interdisciplinary research programme aimed at examining different levels of behavior change in relation to cardiovascular disease, through the study of a long-standing primary care health partnership in one Swedish region. STAR-C aims to study and understand what facilitates or impedes positive behavior change at individual, community and system levels in the context of cardiovascular disease; to introduce a virtual coaching platform to support such individual behavior change; and further to evaluate the virtual coaching platform prospectively.

To date, the use of technology in health promotion, particularly in the primary health care setting, has not received sufficient scientific attention. It is expected that the rollout and scale-up of technological interventions for health promotion will accelerate in the near future, both in Sweden (59) and in other countries (48). Much earlier research on health-related technology has focussed on the technical aspects of care applications (49). Lately though, there have been calls for a broader approach that accounts for the contextual conditions for actual users, like social networks, gender, age, geography, education, etc. (50–53), including social media usage (54).

This research programme will attempt to answer how these technologies can and should be used, and what risks, limitations and benefits may be expected. Here a mixed-method approach helps provide a robust methodological framework for both design and evaluation (55). The interdisciplinary nature of STAR-C, integrating both quantitative and qualitative paradigms and methods as well as knowledge from all our disciplines, will allow the researchers to understand not only the complexity but also the added value and ethical dilemmas involved in integrating digital technology into health promotion and disease prevention. Different qualitative methods will provide an in-depth understanding of the social and cultural contexts where innovations and interventions will take place and will be scaled-up. The approach to (i) map the behavioral trajectories and their impacts on population health, and (ii) use the results as a novel risk prediction model in the behavioral change programme for middle-aged adults are innovative and tailored to different targeted end-users.

STAR-C will not only increase knowledge about how the interaction between health-related technology and social media is comprehended and practiced in users' daily life, but also about any health-enhancing aspects or risks that can be ascribed to the communicative aspects of social media sharing. With specific regard to the VIP, this programme studies how information about the VIP as a whole—including the experience of having the health dialogue that is part of it—and individual results emanating from the VIP, are being (or not being) disseminated in social media. Further, the programme deepens our theoretical conceptions of the interactions between individuals, social media and other technologies, thereby shedding light on the “whats,” “hows,” and “whys” of social media health data sharing. Hence this research programme will contribute to filling gaps of knowledge in the design, implementation, process, and evaluation of such technologies, to promote health, and well-being

A thorough focus on social and cultural contexts, gender, and other axes of inequality plays a central role in STAR-C. Adult populations constitute heterogeneous groups of people with an increasing prevalence of comorbidities of NCDs. Structural level determinants such as political contexts, cultural and societal norms, social position including gender, education, income, and ethnicity are critical root-cause determinants of health and health inequalities among adult populations (56). Despite the higher life expectancy observed among women globally, women experience worse health than men, leading to more of them, especially older women, living with disability (57). But if women have access to care, they adhere better to health messages and treatments than men do, which might be understood due to the somewhat different dynamic of women's social networks. This might influence their propensity to adopt innovation and progression through different stages of behavioral change, when exposed to health promotion programmes (31, 35).

The STAR-C programme will investigate gender inequalities in behavioral and health trajectories. It explores the significance of gender for women and men's risk perception and receptiveness to risk communication, stages of behavioral change, and readiness to adopt new innovations and technologies for behavioral change programmes. We will scrutinize the contradiction in women's higher morbidity despite their better adherence to health messages. Understanding the heterogeneity of the processes and determinants of health in women and men is essential for developing gender-sensitive public policies that will promote health and social well-being for all members of an adult population. A gender-sensitive policy takes into account differences and commonalities of women and men and considers their different circumstances and specific problems (58). The implementation of a comprehensive and gender-sensitive health promotion programme for adult populations may reduce gender gaps in health and longevity.

## Conclusion

The STAR-C research programme will address the knowledge and practice research gaps in the use of information technologies in health promotion and NCD prevention programmes with specific foci on: (1) the development of a technology platform for personalized digital coaching for promoting healthy behavior; (2) exploring the adoption of health technology in different social and cultural contexts for leveraging the impacts of a health programme in the population beyond the healthcare setting; and (3) co-creation of knowledge and close collaboration between researchers, health professionals, and the end users as the beneficiaries of STAR-C.

Through the dissemination of health risks to the wider population, STAR-C expects to contribute to narrowing the health inequality gaps observed between different population groups. The programme will increase the understanding of and promote public engagement in health promotion activities that encourage healthy behaviors among the adult population in target populations. The results of STAR-C also have potential to be tested and scaled up in other countries, including low- and middle-income countries with large and aging populations, where the epidemic of NCDs is growing rapidly and health systems are under heavy constraints. Ultimately, STAR-C supports the Sustainable Development Goals, especially with regard to Goal 3 on good health and well-being.

## Ethics Statement

The studies involving human participants were reviewed and approved by The Swedish Ethical Review Authority (Dnr. 2019-02924;2020-02985). The patients/participants provided their written informed consent to participate in this study.

## Author Contributions

All authors have provided a substantial and critical inputs to the work and approved the final draft for publication.

## Conflict of Interest

The authors declare that the research was conducted in the absence of any commercial or financial relationships that could be construed as a potential conflict of interest.
